# Prophylactic pectoralis major flap to compensate for increased risk of pharyngocutaneous fistula in laryngectomy patients with low skeletal muscle mass (PECTORALIS): study protocol for a randomized controlled trial

**DOI:** 10.1186/s12885-023-11773-7

**Published:** 2024-01-15

**Authors:** Maartje A. van Beers, Caroline M. Speksnijder, Carla H. van Gils, Geert W.J. Frederix, Jan Willem Dankbaar, Remco de Bree

**Affiliations:** 1 Department of Head and Neck Surgical Oncology, University Medical Center Utrecht, Heidelberglaan 100, Utrecht, 3584 CX The Netherlands; 2 Department of Oral and Maxillofacial Surgery and Special Dental Care, University Medical Center Utrecht, Utrecht University, Utrecht, The Netherlands; 3 Julius Center for Health Sciences and Primary Care, University Medical Center Utrecht, Utrecht University, Utrecht, The Netherlands; 4 Department of Radiology, University Medical Center Utrecht, Utrecht University, Utrecht, The Netherlands

**Keywords:** Total laryngectomy, Head and Neck cancer, Low skeletal muscle mass, Sarcopenia, Myofascial pectoral major flap, Pharyngocutaneous fistula

## Abstract

**Background:**

Total laryngectomy (TL) is a surgical procedure commonly performed on patients with advanced laryngeal or hypopharyngeal carcinoma. One of the most common postoperative complications following TL is the development of a pharyngocutaneous fistula (PCF), characterized by a communication between the neopharynx and the skin. PCF can lead to extended hospital stays, delayed oral feeding, and compromised quality of life. The use of a myofascial pectoralis major flap (PMMF) as an onlay technique during pharyngeal closure has shown potential in reducing PCF rates in high risk patients for development of PCF such as patients undergoing TL after chemoradiation and low skeletal muscle mass (SMM). Its impact on various functional outcomes, such as shoulder and neck function, swallowing function, and voice quality, remains less explored. This study aims to investigate the effectiveness of PMMF in reducing PCF rates in patients with low SMM and its potential consequences on patient well-being.

**Methods:**

This multicenter study adopts a randomized clinical trial (RCT) design and is funded by the Dutch Cancer Society. Eligible patients for TL, aged ≥ 18 years, mentally competent, and proficient in Dutch, will be enrolled. One hundred and twenty eight patients with low SMM will be centrally randomized to receive TL with or without PMMF, while those without low SMM will undergo standard TL. Primary outcome measurement involves assessing PCF rates within 30 days post-TL. Secondary objectives include evaluating quality of life, shoulder and neck function, swallowing function, and voice quality using standardized questionnaires and functional tests. Data will be collected through electronic patient records.

**Discussion:**

This study’s significance lies in its exploration of the potential benefits of using PMMF as an onlay technique during pharyngeal closure to reduce PCF rates in TL patients with low SMM. By assessing various functional outcomes, the study aims to provide a comprehensive understanding of the impact of PMMF deployment. The anticipated results will contribute valuable insights into optimizing surgical techniques to enhance patient outcomes and inform future treatment strategies for TL patients.

**Trial registration:**

NL8605, registered on 11-05-2020; International Clinical Trials Registry Platform (ICTRP).

## Introduction

**Fig. 1:**
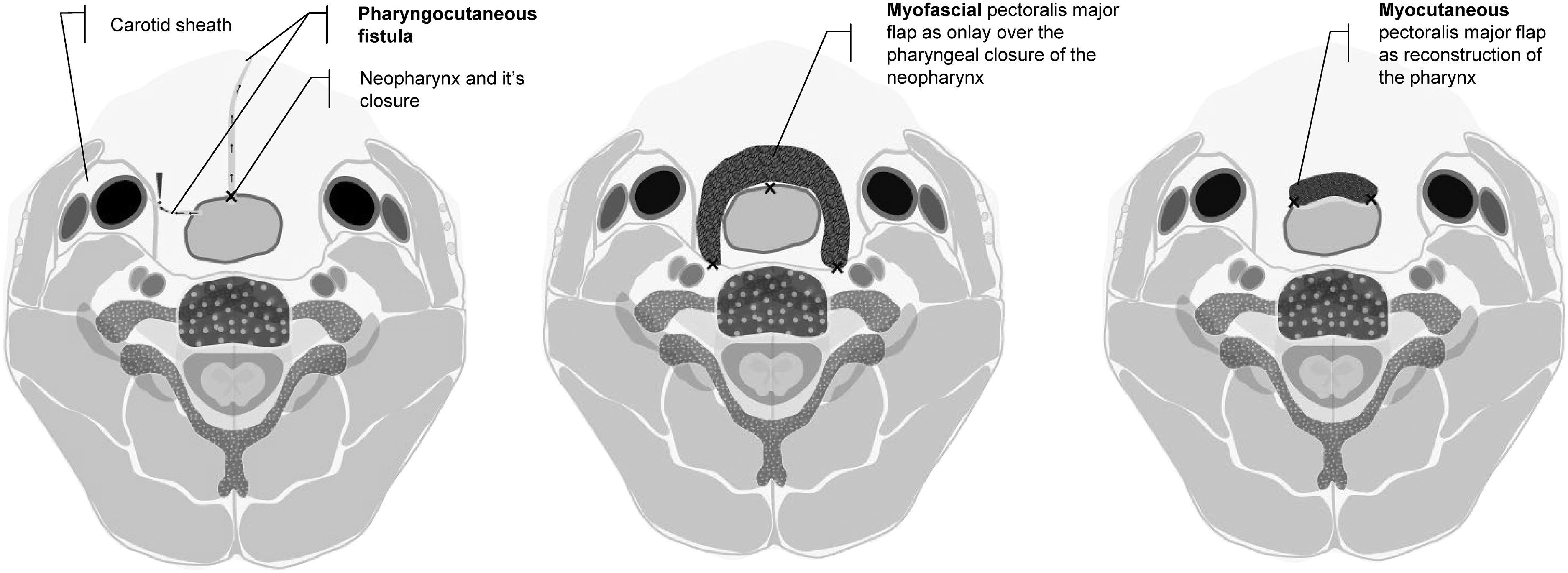
Simplified image of the neopharynx, a pharyngocutaneous fistula, myofascial and myocutaneous pectoralis major flap

Total laryngectomy (TL) is performed routinely in patients with primary advanced laryngeal or hypopharyngeal carcinoma with invasion of the thyroid or cricoid cartilage and/or extra laryngeal soft tissue. TL is also indicated in patients with residual or recurrent disease after treatment with chemoradiation or radiotherapy solely and patients with a dysfunctional larynx due to posttreatment sequalae. During TL, the distinction between the swallowing and breathing pathways is established by forming both a neopharynx and a tracheostoma. A pharyngocutaneous fistula (PCF) is one of the most common postoperative complications after TL and is defined as a saliva-leaking communication between the neopharynx and the skin (see Fig. 1). PCF mostly exists between the mucosal line of the neopharynx and the surgical skin incision or, but less frequently, around the tracheostoma [1, 2]. Incidence rates vary between 6% and 58% in literature [3]. In a nationwide Dutch study an overall incidence rate of 26% in 324 patients undergoing TL was found [4].

PCF is associated with severe consequences such as prolonged hospital stay and delay or interruption of the start of oral feeding and voice rehabilitation, leading to a long healing course significantly impacting the patient’s quality of life [5–7]. In addition, PCF may cause complications such as carotid artery rupture or delay of the needed adjuvant treatment, potentially jeopardizing optimal oncologic treatment [4–8]. PCF has even been associated with an increased risk of distant metastases after TL salvage [9].

Conservative treatment of PCF usually consists of local wound treatment and antibiotics, and the patient is fed by a nasogastric tube or parenteral nutrition. However, due to the breakdown of the mucosal suture and therefore the constant flow of saliva into surrounding soft tissues, wound healing is often impaired. Surgical closure of PCF after failure of the conservative treatment is indicated in 37–58% of the patients [2, 7, 10]. In summary, preventing PCF holds the potential to minimize the influence of the negative outcomes on the patient’s quality of life, help to avoid additional surgeries and their associated morbidity and reduce the risk of life-threatening complications.

One of the surgical strategies to minimize PCF development following TL is the transfer of a myofascial pectoralis major flap (PMMF) to the neck as onlay for reinforcement of the pharyngeal closure (see Fig. 1) [11]. It has been shown that a prophylactic PMMF reduces the risk of PCF in TL patients significantly [12–14] or PCFs were smaller and less likely to require surgical repair [11–15]. Prophylactic PMMF has also demonstrated to decrease a patient’s morbidity and hospital stay and results in financial savings for the healthcare system [16].

Several risk factors for PCF have been described in literature such as prior chemoradiotherapy, the extent of the pharyngectomy, neck dissection, pre-treatment tracheostomy, preoperative albumin and low BMI [3, 4, 17, 18]. A nationwide Dutch study showed a broad range of PCF incidence between the centers of the Dutch Head and Neck Society (NWHHT), which could not be fully explained by the prediction model developed with known risk factors know at that time. More recently also, a preoperative radiological assessed low skeletal muscle mass (SMM) was found to be an independent risk factor for PCF development [18, 19].

In recent years, research on body composition and especially on SMM has increased. It appears that a low SMM is associated with acute and late adverse events in patients with head and neck cancer during (chemo)radiotherapy [20–23], flap-related complications [24, 25], decreased survival rates [26–29], and PCF [18, 19]. This emphasizes the importance of considering SMM in assessing PCF risk.

Therefore, in this randomized controlled trial (RCT), our primary aim is to investigate if the use of PMMF as onlay on the pharyngeal closure for reinforcement will reduce the PCF rate in TL patients with a high risk for PCF because of low SMM.

## Methods and analysis

### Objectives

#### Primary objective

To determine and compare among patients with low SMM, the PCF rate in those with PMMF as onlay for reinforcement to the PCF rate in those without PMMF. PCF rate will also be evaluated in patient without low SMM and in patients who unexpectedly needed the PMMC for reconstruction of the pharynx.

#### Secondary objective(s)

Secondary outcome measurements will only be scored in the group with low SMM. In this group, the following outcomes are compared between the group with and without PMMF using questionnaires and function tests.

- Quality of life.
- Shoulder and neck function.
- Swallowing function and dysphagia complaints.
- Voice quality and it’s psychological consequences.
- Patient’s perspective.
- The healthcare related costs.

### Study design and population

This multicenter PECTORALIS-study is designed as a randomized clinical trial (RCT) and funded by the Dutch Cancer Society (KWF) (NL72319.041.20).

Patients who are planned for TL, will be included in this study when they: (1) have a minimum age of 18 years, (2) are mentally competent and (3) have sufficient knowledge of the Dutch language to be able to give informed consent. Patients will be enrolled by their head and neck surgical oncologist and/or by a researcher after consultation in one of the participating tertiary referral centers of the NWHHT or three Belgian (Dutch speaking) centers. Patients will be excluded for this study when they: (1) will be treated with chemoradiotherapy (with cisplatinum/carboplatin) for a previously diagnosed head and neck carcinoma (HNC), (2) will undergo TL with reconstruction of the pharynx with myocutaneous pectoralis major (PMMC), gastric pull up or jejunal flap, (3) have major CT- or MRI-scan artefacts impeding accurate muscle tissue identification, and (4) have an interval between TL and imaging longer than 2 months.

**Fig. 2:**
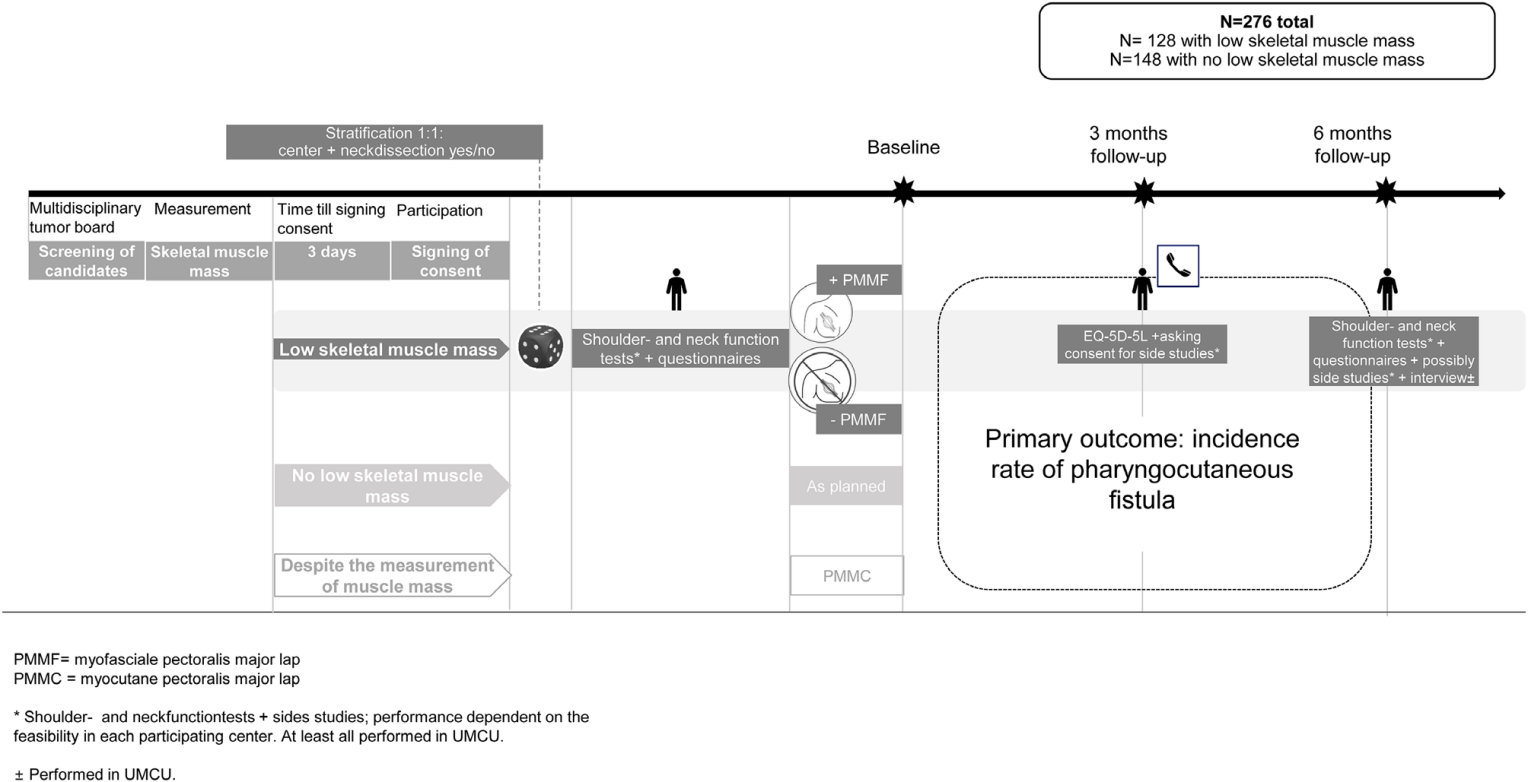
Timeline of including patients

When a patient is eligible for participation in this study, SMM will be measured using routinely performed (FDG-PET/)CT- or MRI scan of the head and neck as described below (see Fig. 2).

After informed consent, patients with low SMM will be centrally randomized between prophylactic PMMF at the time of TL or not. A stratified permuted-block procedure randomizes patients to the groups on a 1:1 ratio. Strata include treating center and concomitant neck dissection. Both primary and secondary outcome measurements as described below will be evaluated in the group with low SMM.

Patients without low SMM will undergo the TL as regularly scheduled, will not be randomized and only the primary outcome measurement will be evaluated.

Patients definitively scheduled for TL with reconstruction of the pharynx using the PMMC meet the exclusion criteria and thus will not be recruited for the study. If in an included patient, regardless of SMM and possible randomization, it is unexpectedly decided peroperatively that a PMMC is required for reconstruction of the pharynx, these patients will be followed over time. The primary outcome measurement will still be evaluated.

In conclusion, the primary outcome measure is thus evaluated in the following groups:

- Patients with a low SMM who will undergo a TL with PMMF.
- Patients with a low SMM who will undergo a TL without PMMF.
- Patients without a low SMM who will undergo TL as regularly scheduled.
- Patients who unexpectedly need a PMMC for reconstruction of the pharynx during the TL, regardless of their SMM.

### Measurement of SMM

The cross-sectional area (CSA) of the paravertebral muscles and both sternocleidomastoid muscles at the level of the third cervical vertebra (C3) will be measured by using (FDG-PET/)CT or MRI. When possible, CT is preferred over MRI because you are aided in accurately delineating the CSA by setting the radiodensity to -29 and + 150 Hounsfield Units (HU) which is specific for muscle mass [30, 31]. If MRI-imaging is used, SMM will be manually delineated, excluding fatty mass through manual means. If FDG-PET/CT is available, SMM will also be measured (directly) at the level of the third lumbar vertebra (L3). The single axial slide at level C3 of imaging which will show both the transverse processes and the entire vertebral arch scrolling from cranially to caudally will be selected. This segmentation of SMM will be performed using the software package SliceOmatic (Tomovision, Canada). CSA at level of C3 will be converted to the CSA at L3 by using the formula as previously described by Swartz et al [30] Then the CSA at L3 will be corrected for height thus creating the lumbar skeletal muscle index (LSMI). A LSMI of ≤ 43.2 cm2 /m2 will be considered as low SMM.

### Intervention

First the neopharynx will be closed. The PMMF will be harvested by elevating the muscle off the chest like the myocutaneous pectoralis major (PMMC) flap, but without the skin and subcutaneous fat of the donorsite (see Fig. 1). Then the muscle and its fascia will be tunneled into the neck and sutured to different structures around the neopharynx. In this manner, the PMMF will be used as a muscular vascularized flap and additional layer to cover the delicate closure of the neopharynx [11, 32].

### Outcome measurements

#### Primary outcome measurement

As mentioned above, the PCF-rate following TL will be scored in patients with a low SMM who will undergo a TL with or without PMMF, without low SMM (undergoing TL as regularly scheduled) and in patients who unexpectedly need a PMMC for reconstruction of the pharynx during the TL, regardless of their SMM.

PCF is defined as a clinical fistula requiring any form of conservative or surgical treatment occurring within 30 days after TL. To also assess the prevention of possible PCF development, the results of the swallow X-ray and their potential impact on the patient’s oral intake are taken into account. This approach aims to obtain the most comprehensive evaluation of PCF incidence.

#### Secondary outcome measurements

In low SMM patients shoulder and neck function, swallowing function, and voice quality with their consequences on quality of life (QoL) will be investigated by questionnaires before and 6 months after TL.

The following questionnaires will be assessed:

- Quality of life: European Organization for Research and Treatment for Cancer Quality of Life Questionnaires, EORTC QLQ-C30, EORTC-QLQ-H&N35, and EuroQol 5D 5 L (EQ-5D-5 L) [33–35].
- Shoulder and neck function: the Shoulder Disability Questionnaire (SDQ) [36], Shoulder Pain and Disability Index Dutch Language Version (SPADI-DLV) [37] and the Neck Disability Index (NDI) [38].
- Swallowing function and dysphagia: The Dysphagia Severity Scale (DSS) and Dysphagia Quality of Life Scale (DQOL), after laryngectomy the Swallow Outcomes After Laryngectomy (SOAL) [39], the M.D. Anderson Dysphagia Inventory (MDADI) [40] and the Functional Oral Intake Scale (FOIS) for dysphagia (the only investigator reported outcome measurement (IROM)) [41].
- Voice quality: Voice Handicap Index (VHI) [42].

Shoulder and neck function tests will be performed depending on the feasibility in the participating center also before and 6 months after TL. In addition, this latter group of patients will be recruited 3 months after TL to have a voice recording and a video fluoroscopy (VFSS). Performance of these side studies will also be performed on the available logistics of the participating center.

### Shoulder and neck function tests

AROM of the shoulders and neck will be performed in the patients’ group with a low skeletal muscle mass before and 6 months after TL according to a standardized protocol. The flexion, abduction, rotation, extension and flexion of the shoulder and neck and forward flexion and abduction the shoulder will be examined using a goniometer. The mean of two sequential measurements will be used for further analysis [43].

### Patients’ experienced need for neck and shoulder rehabilitation

Qualitative research will be performed by semi-structured interviews to get insight in the patients’ experiences with and insights in the treatment and its morbidity, such as the effects on shoulder and neck function, related to provided information and therapy. Data will be analyzed with a thematic analysis approach [44]. This part of the study will be performed and written according to the Standards for Reporting Qualitative Research (SRQR) [45]. Participants will be recruited until saturation will be achieved, which is when no new information will be identified from the last two interviews and expected to occur between six and twelve interviews [46, 47].

The semi-structured interviews will be conducted using pre-defined topic guides. This topic guide is open to changes when interviews identify new information. All participants will be asked about possible shoulder and neck function problems, how this is handled by the patient and whether rehabilitation was required.

### Swallowing function

Function tests on the swallowing quality of the TL-patients with low SMM will be assessed by the performance of videofluoroscopy (VFSS). Patients will be offered thin liquid (thinned Micropaque), thick liquid (Micropaque purely) and firm consistency (toast in Micropaque) in 3 steps. Each step will be performed twice.

### Voice quality

The quality of the voice of patients with low SMM will be measured by the performance for voice recording and the associated Acoustic Voice Quality Index (AVQI) [48, 49]. AVQI is a multi-parameter model in which the outcomes of six acoustic parameters are measured and combined into one objective measure of the voice quality.

### Other parameters

Patients’ demographic, staging, treatment and outcome data will be collected using electronic patient records. To allow for comparison with the recent Dutch Head and Neck Society audit the same characteristics and potential predictive factors will be evaluated [4]. The following parameters will be added: peroperative data (i.e. type of closure of the neopharynx), comorbidity scores (ACE-27 and Charlson Comorbidity Index), American Society of Anesthesiologist’s physical status (ASA score), WHO performance status and preoperative laboratory results, which will be analyzed from routine blood tests. General postoperative complications (except from PCF-rate) are graded according to the Clavien-Dindo classification of Surgical Complications [50]. Severe complications are defined as Clavien-Dindo grade 3 A or higher [41–44].

### Cost-effectiveness analysis

A detailed analyses of cost and effect differences for patients having a PMMF and standard of care (no PMMF) will be assessed using a health care perspective. All healthcare consumption for every individual patient will be collected from electronic patient files. Subsequently units of health care consumption will be linked to respective Dutch unit costs according to available lists of the Dutch Health Care Institute. The economic evaluation will take place both via a trial based approach and making use of decision analytical modeling to extrapolate outcomes. Uncertainty of outcomes will be depicted by both deterministic as well as probabilistic sensitivity analyses.

### Power calculation

Subtraction of data from the meta-analyses from Paleri et al. [13] and Sayles et al. [12] revealed that the PCF rate for patients with and without PMMC or PMMF for reinforcement is reduced (11/114 (0.10) to 47/156 (0.30)), giving a relative risk of 0.32. After exclusion of the patients who received a reconstruction of the pharynx from the database of Bril et al. [18], the PCF rate in patients with low SMM was 31.0%. Assuming that the same relative risk as in the meta-analyses is applicable, this leads to our hypothesis that a prophylactic PMMF can reduce the PCF rate from 31.0 to 9.9%.

To show that the use of PMMF can reduce the fistula rate for TL patients with low SMM, 61 patients per arm are needed (two sided alpha 0.05 and power 85%). With an expected drop-out of 5%, a total of 128 patients with low SMM are needed. This power calculation was performed with the program PASS (two-sided Z-test with pooled variance). Since approximately 46% of TL patients has low SMM, a total of about 276 TL patients are required to include 128 patients with low skeletal muscle mass.

### Statistical analysis

Our primary hypothesis is that the use of PMMF as onlay for reinforcement can reduce the PCF rate in patients with low SMM after TL from 31.0 to 9.9%. To test this hypothesis, we will compare the incidence of fistula formation in patients with low SMM between the group with PMMF (intervention arm) and the group without PMMF lap (control arm) by the Chi-squared test or when needed the Fisher’s exact test (N < 5). To demonstrate the association between SMM and fistula formation, the incidence of fistula formation in the control arm (low SMM without PMMF) will be compared with the incidence of fistula formation in the (non-randomized) group of normal SMM. The relative risk will be calculated with an associated 95% confidence interval. Modified Poisson regression models will be used to correct for potential confounder, such as radiotherapy in prehistory, type of closure of the neopharynx etc.

Results of our other outcomes will be presented as the mean scores with standard deviation for continuous variables or as median with interquartile range for ordinal or non-normal distributed continuous data. Differences between groups with or without PMMF will be tested by independent t-tests for normally distributed continuous data and for ordinal and non-normal distributed continuous data Mann Witney U tests will be used. Differences over time within groups with or without PMMF will be tested by paired t-tests for normally distributed continuous data and for ordinal and non-normal distributed continuous data Wilcoxon signed-rank tests will be used.

### Analyses of semi-structured interviews

Semi-structured interviews will be analyzed by two researchers using thematic descriptive analyses [44]. This thematic analysis will be an independent qualitative descriptive approach to identify, analyze and report patterns (themes) within the data. Data analysis will be performed by two researchers independently and compared after the third and last interview when saturation is reached. During analysis we will search for the identification of common threads that extend across the interviews. This will provide a detailed, and nuanced account of data by breaking the interview texts into relatively small units. Practically the semi-structured interviews will be transcribed verbatim, anonymized and will be thoroughly read several times. Thereafter initial codes will be generated, followed by the search for themes, reviewing these themes and finally defining and naming the themes. These themes will be reported and will be supported by compelling extract examples relating back to the analysis to answer the research question. Quotes from the interviews will be used to support the themes. All quotes provided in the article will be translated into English.

## Discussion

Skeletal muscle mass (SMM) has emerged as a critical predictive factor for various adverse outcomes following medical interventions. For instance, in patients with HNC undergoing treatment, a low SMM has been identified as a significant risk factor for adverse events, such as PCF development subsequent to TL. Given the undesirable nature of PCF, proactive identification of individuals at risk becomes imperative. Notably, patients previously subjected to CRT for HNC cancer have an elevated risk of PCF development and generally receive routinely PMMF reinforcement during TL. Hence, the aim of this trial is to assess whether utilizing PMMF as an onlay technique for pharyngeal closure reinforcement can effectively reduce PCF incidence among high-risk TL patients with low SMM.

Numerous techniques are available for evaluating body composition and SMM. These methodologies encompass DEXA-scans, BIA, and imaging modalities like CT and MRI. Among these, the measurement of CSA at the level of L3 on CT scans has gained prominence due to its strong correlation with total skeletal muscle volume. To account for individual height variations, CSA is normalized using squared height, resulting in the calculation of skeletal muscle index (SMI; cm²/m²). Recognizing the limited availability of abdominal CT scans in HNC patients, a novel approach for SMM assessment utilizing a single CT slice at the level of C3 was introduced by Swartz et al. [30]. This method exhibits robust correlations with L3 CSA measurements, further enhanced by a multivariate formula that predicts L3 CSA based on C3 CSA, gender, age, and weight. This method is validated [51] with a very good interobserver agreement and intraobserver agreement [52, 53]. CSA can be measured on the level of C2, C3 and C4 and all showed a very strong and significant correlation with the SMI at the level of L3 [54]. However, the most effective discriminator for sarcopenia remained the level of C3 for both males and females [54], in some cases dependent on the type of HNC [55]. Measurement of CSA can be performed on CT and MRI interchangeably [52, 56]. The existing methodologies enable straightforward SMM assessments using routine CT or MRI scans during HNC diagnosis and treatment evaluation. Potential influences of variables on SMM measurements like contrast usage and slice thickness in CT scans [53, 57] have been explored or are currently being investigated (to be published). The clinical relevance of small detected differences in CSA measurements will also be assessed in this research.

This study excludes patients undergoing pharyngeal reconstruction with PMMC or gastric pull up and jejunal interponate. Patients who undergo TL with gastric pull-up reconstruction or jejunal interponate frequently undergo omentum overlay as well, which functions similarly to a PMMF. This introduces a potential bias into the study results and therefore these patients will be excluded.

An inherent challenge of this study pertains to defining the primary outcome measurement, the PCF. The study’s PCF definition entails a clinically diagnosed communication between the neopharynx and the outside of the skin within 30 days after TL. In general, many centers perform a protocol-mandated barium swallow X-ray 7 or 10 days postoperative. In cases where contrast leakage is detected during the swallow X-ray, a one-week delay in initiating oral intake is implemented to mitigate the potential formation of a fistula. Nevertheless, the routine performance of a swallow X-ray varies across the participating centers in this study, complicating the comparison of PCF incidence rates. To address this challenge, a questionnaire survey was conducted to assess variations in protocols related to the prevention, diagnosis or definition, and treatment of PCF among different centers within the NWHHT (to be published). Based on these results we will collect all data on the diagnosis and development of PCF and affecting factors. This encompasses whether a clinical PCF developed within 30 days post TL, a swallow X-ray was conducted according to protocol or due to other considerations, if methylene blue is used or drain fluid is tested for amylase for diagnosis of PCF and the timing of oral intake initiation. By adopting this approach, we aim to score our primary outcome measure as completely as possible.

The secondary outcome measures encompass the impact of PMMF deployment on a range of factors, including QoL, shoulder and neck function, swallowing function, and voice quality. The harvest of the PMMF might influence shoulder and neck function, as the pectoralis major (PM) flap contributes to movement of mainly the shoulders [58, 59]. The neck and shoulder morbidity seems not to be increased by PMMF when patients already underwent a neck dissection [60]. In our study, in addition to specific questionnaires, we will also perform function tests by measuring the AROM of the shoulder and neck before and after surgery. This will allow data to be compared both within the patient (before and 6 months after TL) and between patients (patients with low SMM and TL with and without the PMMF), thus providing the fullest possible representation of the effects of the PMMF on these functions.

Function tests will also be performed (at the ability of the participating center) on the patient’s swallowing function and voice quality. The effect of the PMMF on swallowing function and voice quality is not yet fully understood. In particular, some studies describe a possible effect on swallowing function because of the bulkiness of a myocutaneous PM-flap [61]. However, this flap contains both skin and subcutaneous fat which significantly increases the thickness compared to the myofascial PM-lap as used in this study. Possible effects of PMMF on the voice quality are not explored extensively yet. Jacobi et al. assessed surgical parameters correlating with voice quality [62]. The standard TL was compared to TL with PMMF for reinforcement (n = 10). Speech and voice measures were comparable in both groups. This means that an impact on voice quality of the reconstruction with the PMMF is not expected, but cannot be completely ruled out. Therefore, in addition to administering questionnaires on these functions, we also perform function tests.

In conclusion, this study endeavors to shed light on the efficacy of PMMF deployment as an onlay technique for reducing PCF rates among high-risk TL patients with low SMM. Also potential side-effects, e.g. shoulder morbidity, dysphagia and decreased voice quality, will be investigated to allow weighing the advantages and disadvantages of the use of the PMMF as onlay technique in the management of TL patients. With this study we hope to be able to answer the question whether patients with low SMM, and therefore a high risk of PCF development, should receive PMMF during TL as standard in the future.

## Data Availability

Not applicable.

## Abbreviations

ASA: American Society of Anesthesiologist’s physical status
AROM: Active range of motion
AVQI: Acoustic voice quality index
CSA: Cross-sectional area
CRT: Chemoradiotherapy
DSS: Dysphagia Severity Scale
DQOL: Dysphagia Quality of Life Scale
EQ: EuroQol
EORTC QLQ: European Organization for Research and Treatment for Cancer Quality of Life Questionnaires
FOIS: Functional Oral Intake Scale
HNC: Head and Neck Cancer
IC: Informed Consent
IROM: Investigator reported outcome measurement
HU: Hounsefield Units
LSMI: Lumbar skeletal muscle index
MDADI: M.D. Anderson Dypshagie Inventory
MERC: Medical research ethics committee; in Dutch:medisch ethische toetsing commissie (METC)
NDI: Neck Disability Index
NLR: Neutrophil- lymphocyte ratio
NWHHT: Dutch Head and Neck Society, in Dutch Nederlandse Werkgroep Hoofd-hals Tumoren (NWHHT)
PCF: Pharyngocuteaneous fistula
PMMC: Pectoralis myocutaneous flap
PMMF: Pectoralis myofascial flap
RCT: Randomized controlled trial
SDQ: Shoulder Disability Questionnaire
SMM: Skeletal Muscle Mass
SOAL: Swallow Outcomes After Laryngectomy
SPADI-DLV: Shoulder Pain and Disability Index Dutch Language Version
SRQR: Standards for Reporting Qualitative Research
TL: Total laryngectomy
VFSS: Video Fluoroscopy Swallowing Study
VHI: Voice Handicap Index
